# Supplementary material for: Effects of Various Marine Toxins on the Mouse Intestine Organoid Model
Source: Toxins (Basel). 2022 Nov 24;14(12):829. doi: 10.3390/toxins14120829 (PMC9784231; doi:10.3390/toxins14120829)
Supplement: Supplementary file 1 [file toxins-14-00829-s001.zip › toxins-1923295-supplementary.pdf]

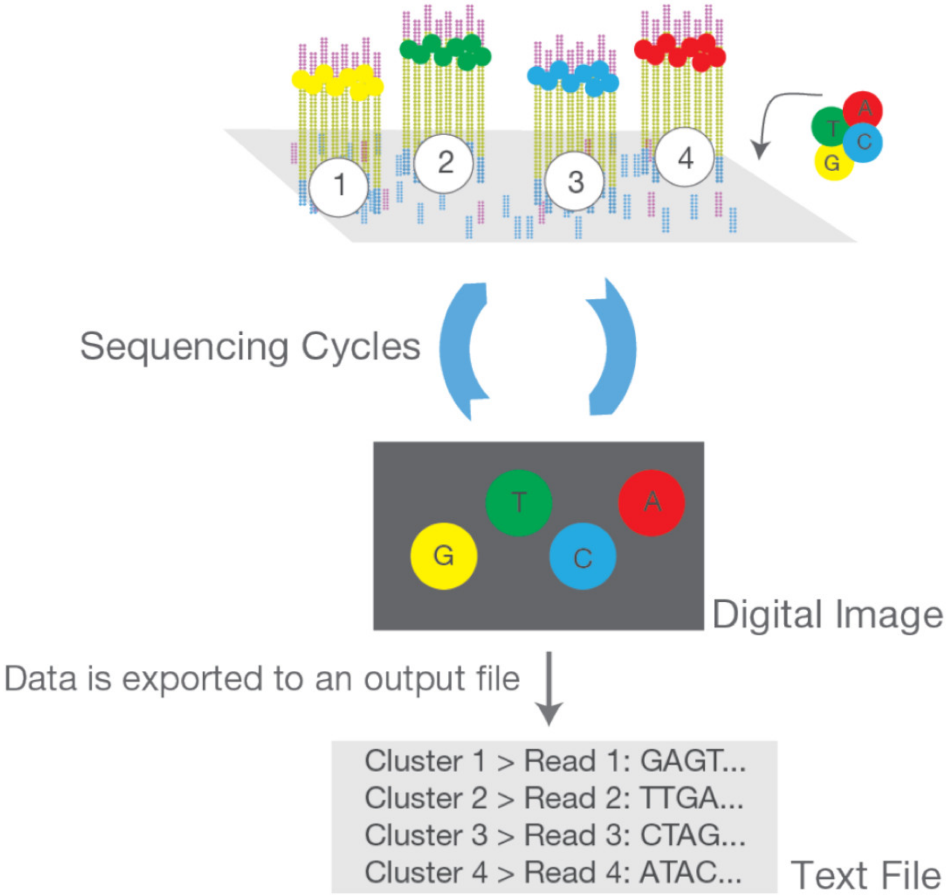

Figure S1. Sequencing process flow chart.

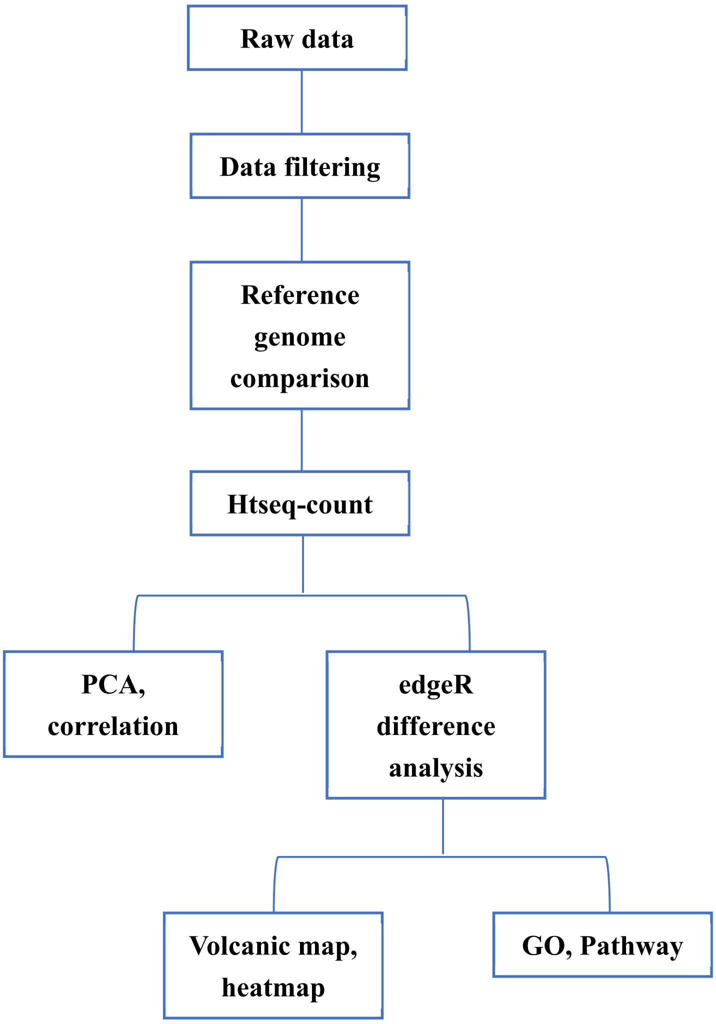

Figure S2. Data analysis flow chart. The analysis process of RNA-seq raw data.

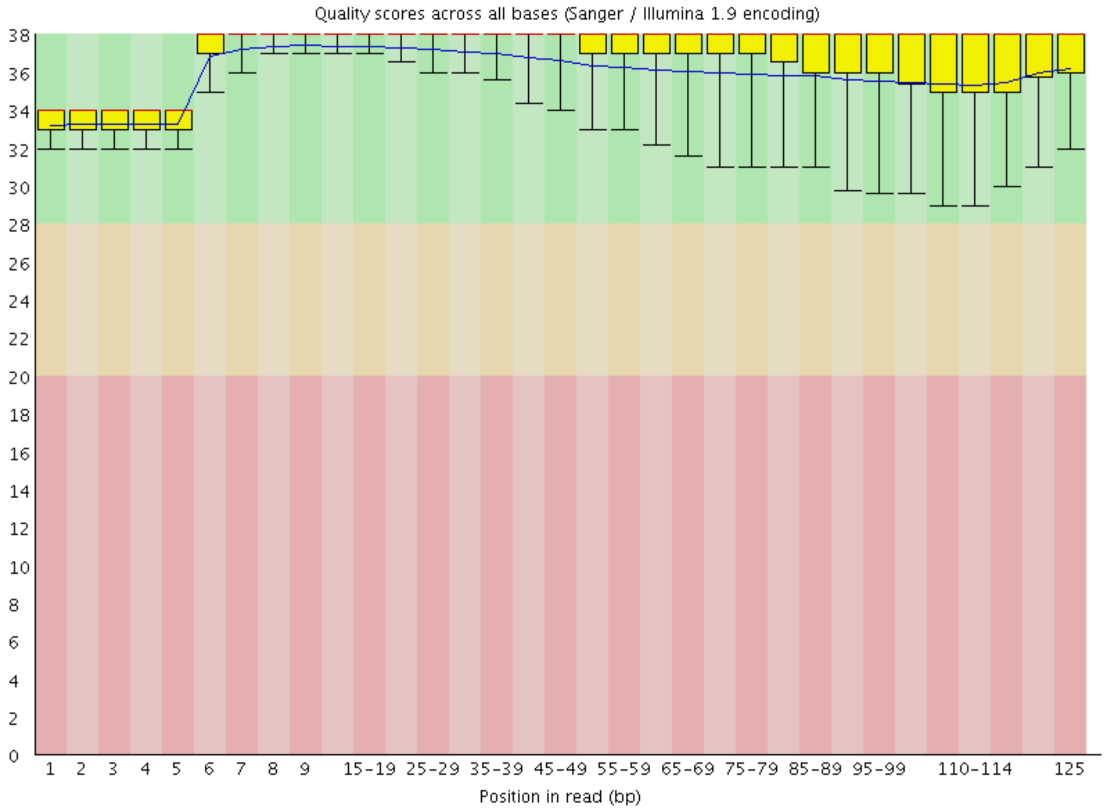

Figure S3. Data Q-value box chart (Box-plot): a box chart is a statistical chart purposed to show the distribution of data. The Q-value box chart is the quality quantile chart. The lowest edge of the yellow rectangle is the Q-value 1/4 quantile, while the upper and lower black lines account for 3/4 of the corresponding quality values, respectively. The blue lines represent the average value of the mass. Different background colors indicate the quality of this part, the green background represents the high quality value part, the orange background indicates the reasonable quality value part, and the red background represents the low quality value part.

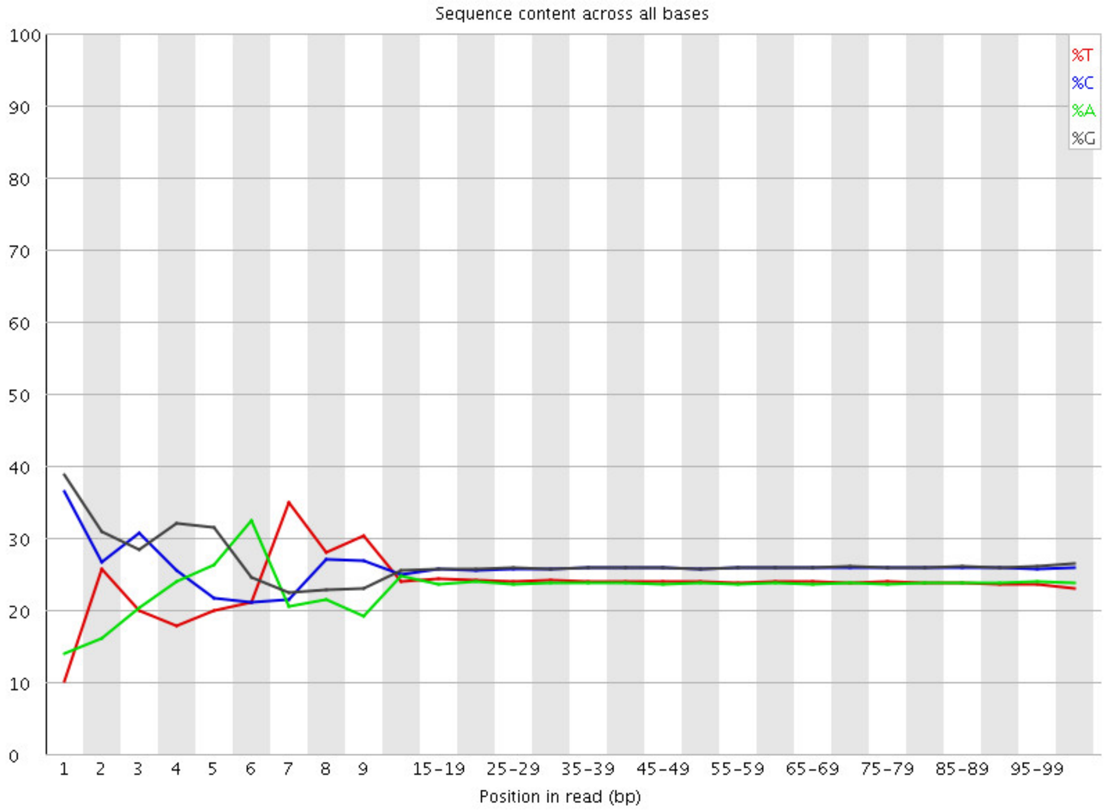

Figure S4. Base distribution map: according to the principle of base complementary pairing, GC and AT base pairs conform to uniform distribution, and the base distribution balance is evaluated against the base distribution map. Due to the random primers used to construct the library, the first few bases will show preference in nucleotide composition to some extent, thus resulting in normal fluctuations. Then, it tends to stabilize.

```
@ST-E00206:377:H5MFVALXX:2:1101:16225:2065
CTGCCCCTTCTGGAGGACCATGAACACTTTATCCCCTGCCAGGGCTTGGAAGTACTCTTCTGTATCTACAGTTGTGCCATCTTCCTCCAGCACCAGGAAGAAGGGCTTGTCTGCCAGCATCAGAGTGTCCCGGACCTTGAGGAGGAGGTC
+
AAFFFKKKKKKKFKK,FKKKKKAFKKKKAKKKKKKKFKFKKKKKKKKKKKAKKKKKKKKKKKK,AA,<FFAK,7FKKKKKKKKKKKKKKKKFKKKKK7KKKF7AKKKFKAKKK7KKKF7<FFKKKFKKK77AFA,<KFF7<<77AAA<AA
@ST-E00206:377:H5MFVALXX:2:1101:17767:2065
CACAGGTAAAATACAGTCGAGAGATTTTCTTGGACTTTAATTCCTTAAATCAGGATAGCAAATAATAGGGGCTTTTACAAATTCAATCTGTTTCCTTACAAAAATTTTCAGCAAAGTAATTTCAGCAAATTAATTTTCAGCAAAGTAAAT
+
AAFFFKKKKKKKKKKKKKKKKKKKKKKKAKKKKKKKKKKKKKKKKKKKKKKKKKKKKKFKKKKKKKKKKKKKKKFKKKKKKKKKKKKKKKKKKKKKKKKKKKKKKKKKKKKKKKKKKKKKKKKKKKKKFFKKKKKKKKKKKFFFFFKKKKFKFK
@ST-E00206:377:H5MFVALXX:2:1101:18052:2065
AGAAAGCAAAGTGTAAGTTTGGAAGAAGGCATGTGCGGTGGTGGCGAGCGCTGCGGGCTATGGAAGGTGACTTGGCTTTTCAGTTAGGGGTAGTGGAGAAAGGGGTAGTTGCTTGTAAACGAGTTAAAAGCATTCAGTGTGGAGGTGTTA
+
AAFFFKKKKKKAKKKAFKKKFK7FKKKKFFKKKKKKKF7<KKKKAFKKKKKKKKKFKKFKKKKKKK7<F7,,FFFAKKKKKKKFFKKKKKKFKKKKKKKAKKKAKFKA<FFFFAKAKKKFKFK,<KFKFFKKFFAAFAFAFKKKKFKKKK
@ST-E00206:377:H5MFVALXX:2:1101:18255:2065
CGAATTCGGGCTTGAGGGAAGCACCACCCACAAGGAAGCCATCCACATCAGGCTGGCTGGCCAGCTCCTTGCAGGTTGCCCCAGTCACAGAGCCTCCATAAATGATACGGGTGCTCTGAGCCACCGCATCAGAGACGTTGGACTTCAGCC
+
AAFFFKKKKKK<FKKFAKKKAKKKKKKK7FKKKKKKKK<AKKKKKKKKKKFKKKKFKKKKKKKFK<,7<FKKKKKKKKKFKF<<FKKKKKK,AAAKKKKKKK7KKKKK7FKKKKKKK,<FFKKAAKKKFKKKKKKKKKK7FKKKKFFFK7
@ST-E00206:377:H5MFVALXX:2:1101:25015:2065
CCTCGAGCCATCGCTACCGCCCTTCGGAACCAGTGCAGCGGCCGATCAGTAAACACAGAGACTGGGGATCGATCATGGGGCTTTGTAAGTGCCCCAAGAGAAAGGTGACCAACCTGTTCTGCTTCGAACATCGGGTCAACGTCTGCGAGC
+
AAFFFKKKKKKKKKKFKKKKFKKKKKKAKKKKKKKKKKKKKFFKKKFKKKAKKKAKKKKKKFKFK<(,7F<FKKKFKKKKKKK7KFKKKKFAKKKKFKKK7KKKKKAKKKKKKFFAKAKKAKKFKFKKKK7AFKFAFF,A77FAKKAFFA
```

Figure S5. Fastq reads chart. Stored in fastq format, the result includes the base composition information of the sequence and its corresponding information about sequence quality. Double-ended sequencing (PE) is divided into two Reads files: _ R1MagneR2.

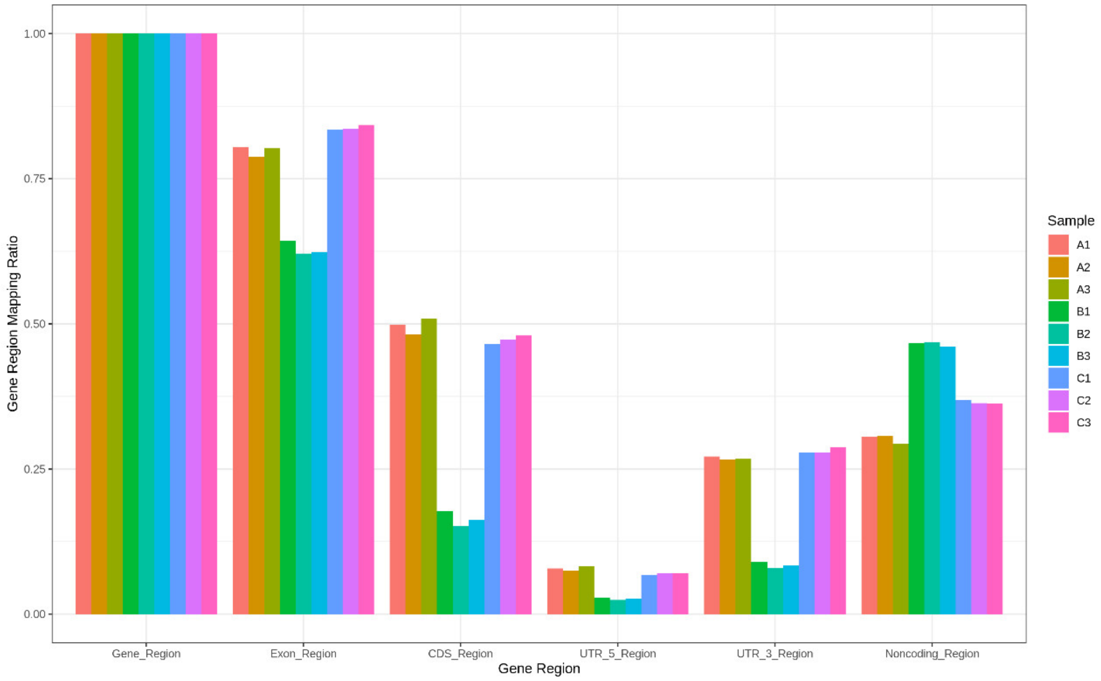

Figure S6. Mapping region comparison of regional distribution. The distribution to different regions of the genome, including gene region, exon region, coding region, 5'UTR region, 3 'UTR region and non-coding region.

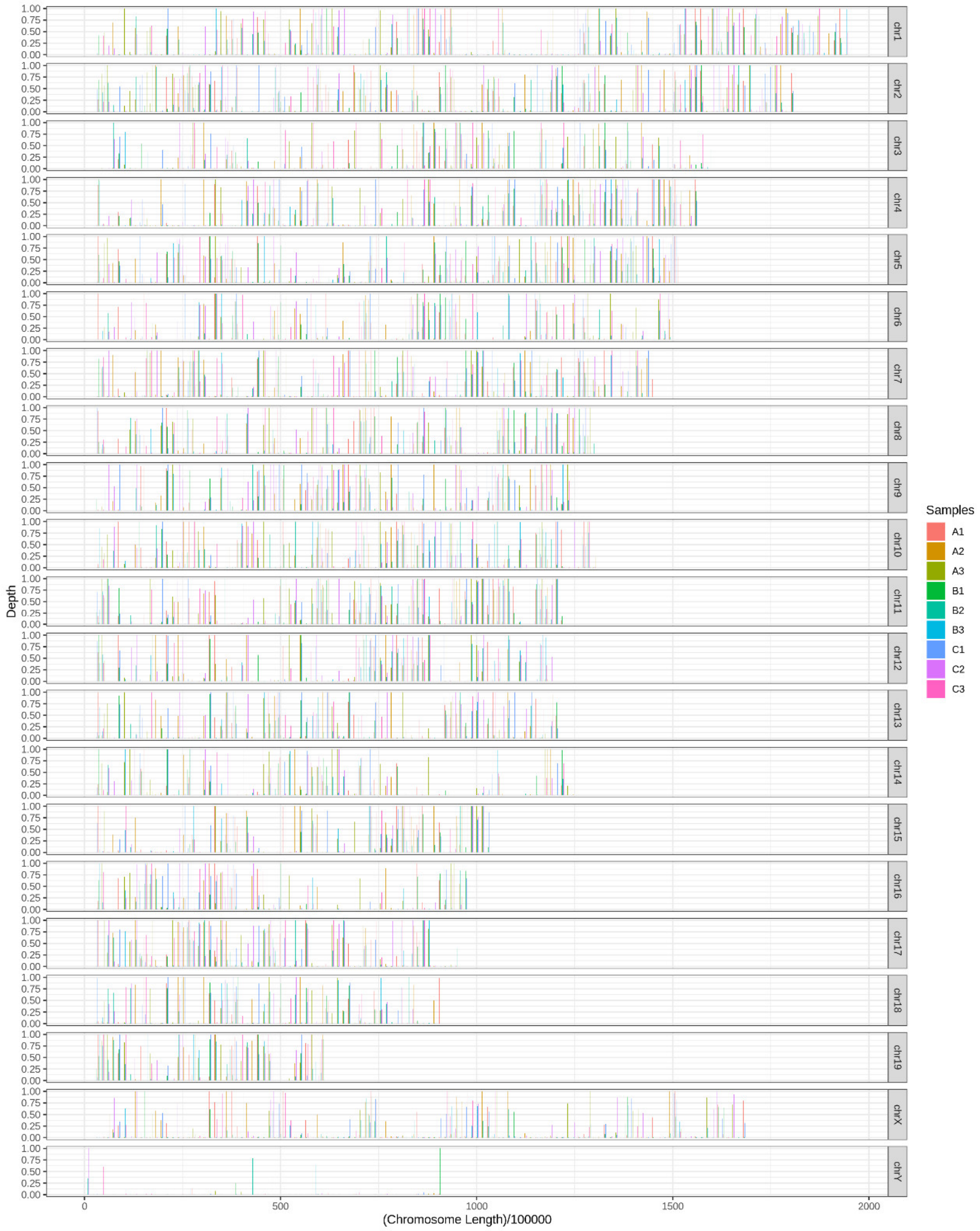

Figure S7. Mapping region genome coverage map. Genome coverage map uses bedtools to calculate the average sequencing coverage and depth of the sequence.

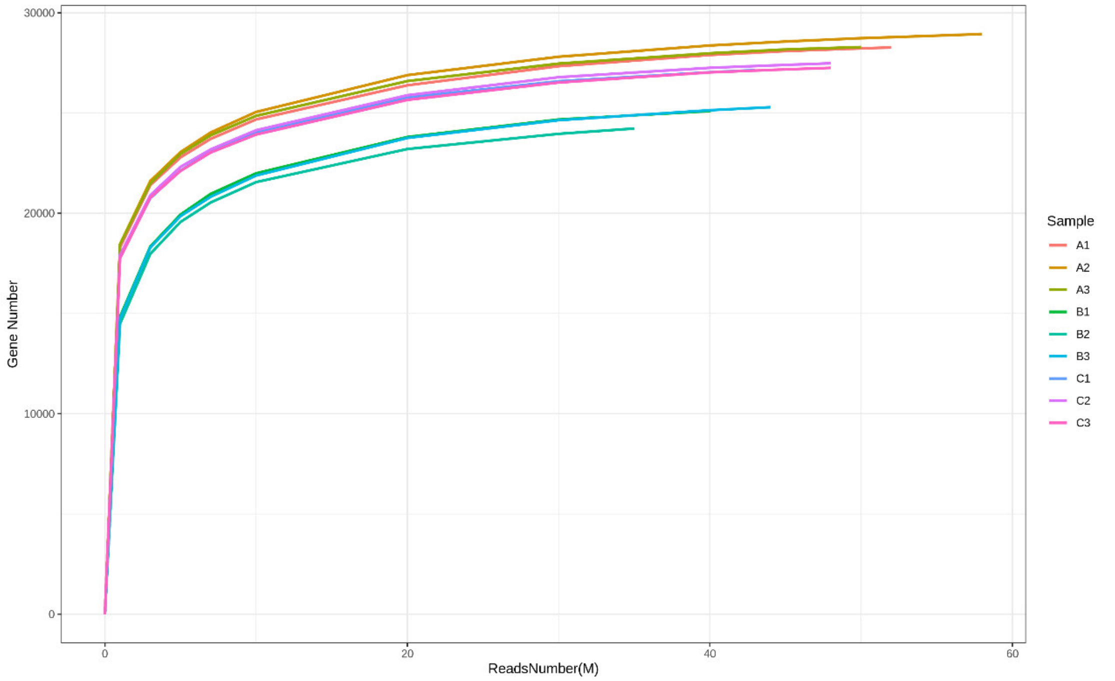

Figure S8. Mapping region saturation analysis. According to the result of data analysis, the number of sequencing (the number of reads) and genome coverage increases exponentially, and then a certain proportion is approached to show saturation. This phenomenon was revealed by saturation analysis. In general, saturation analysis can be conducted to evaluate whether the amount of sequencing data is sufficient.

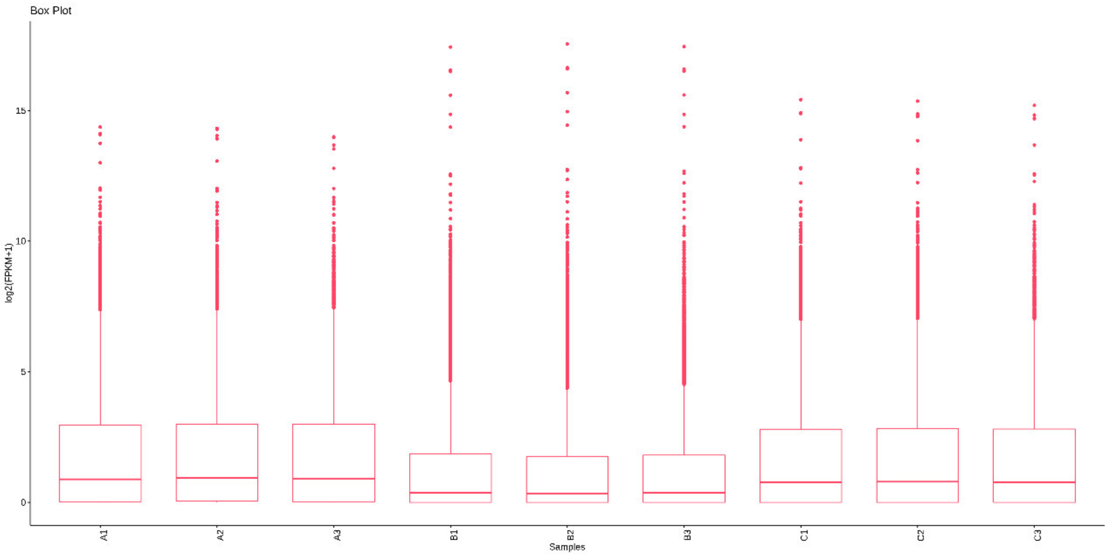

Figure S9. Gene expression boxplot. The box map of all gene expression values (FPKM) of each sample was built to show the distribution of gene expression levels in different samples, where the Abscissa is the sample name and the ordinate is log2 (FPKM+1). The box chart is composed of quartile, the maximum, the upper quartile, the median, the lower quartile and the minimum from top to bottom. The two ends of the box diagram rectangle box correspond to the upper and lower quartile (Q3 and Q1) respectively. The internal median corresponds to the median line, the upper line is Q1+1.5IQR, the underline is Q3-1.5IQR, where IQR (InterQuantileRange) represents the middle quartile range, and

IQR=Q3-Q1.

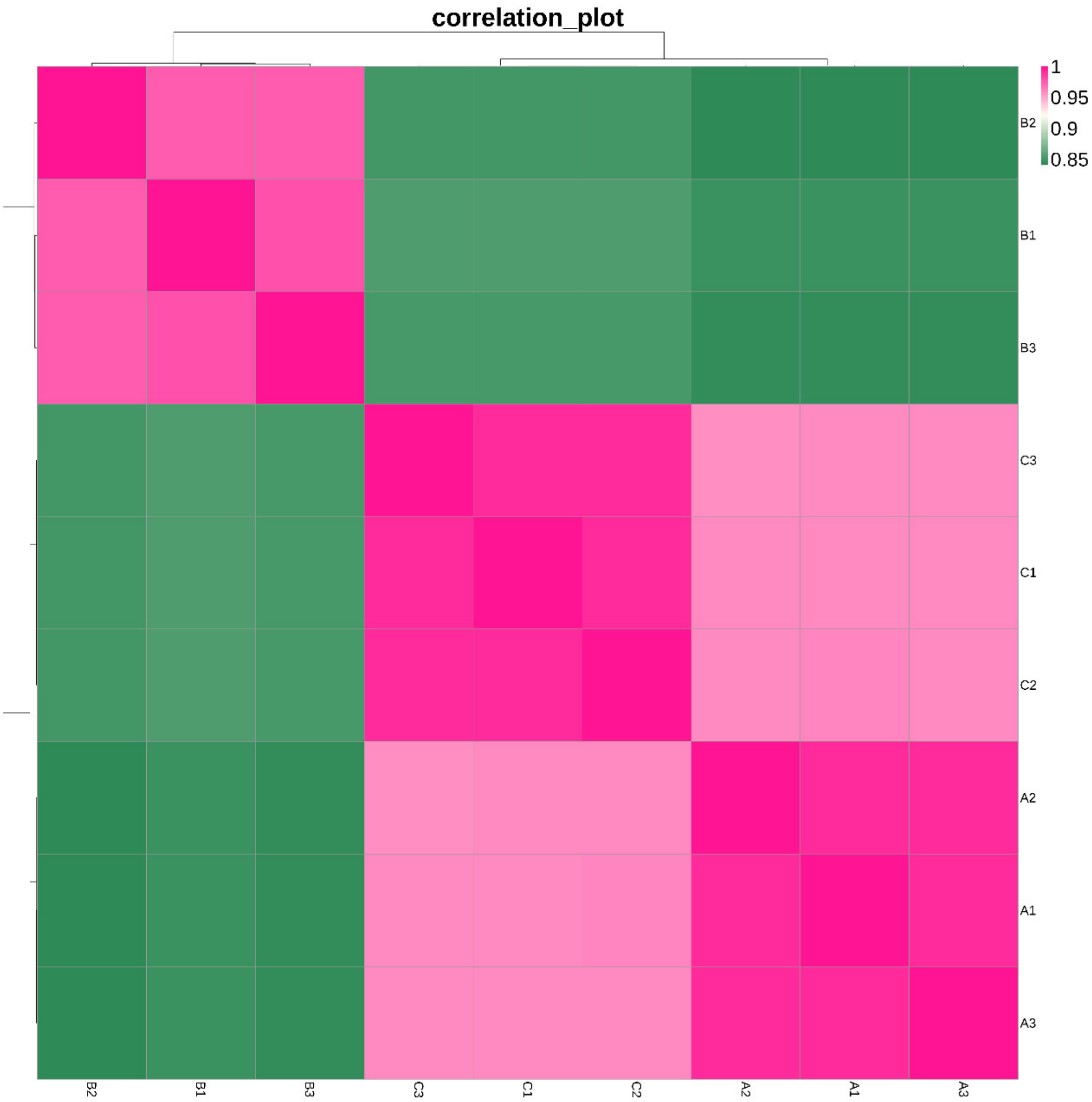

Figure S10. Correlation. The peason correlation coefficient of each sample was calculated and visualized to show the repeatability between groups, such as the log2 processing of data standardized.

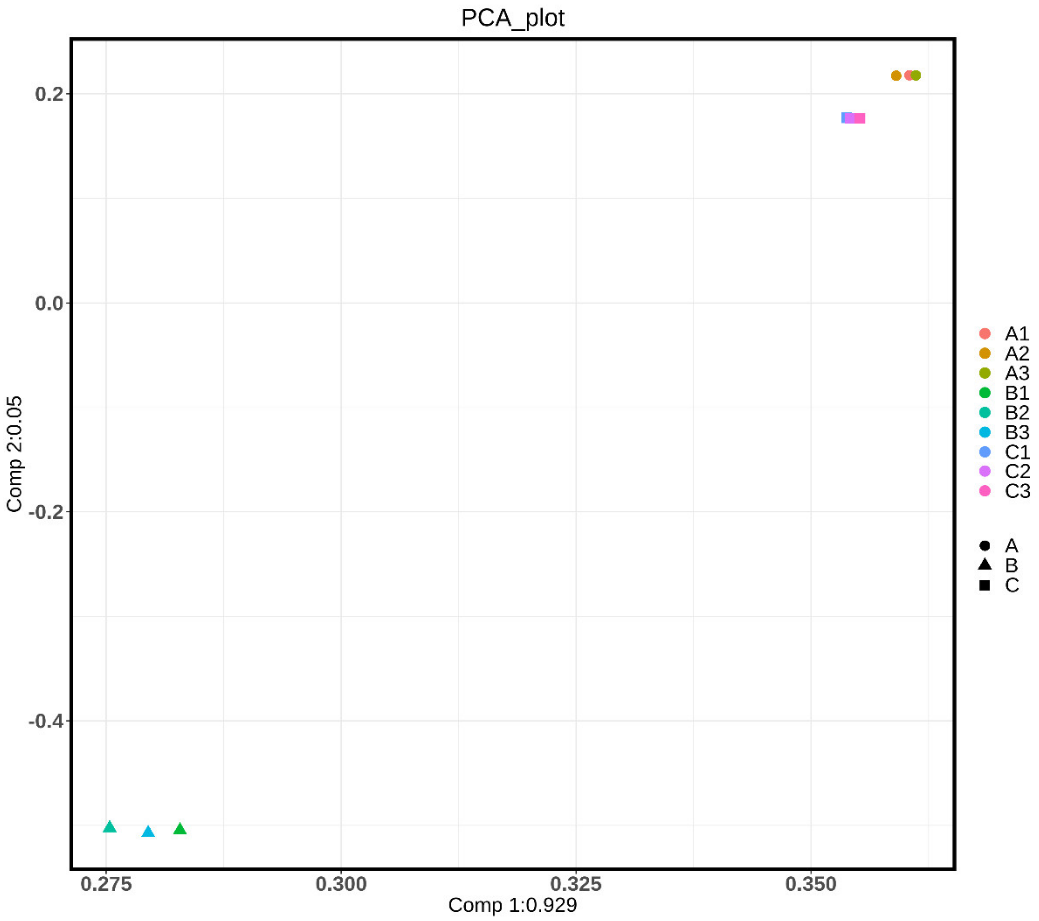

Figure S11. PCA. Based on FPKM method, the dimensionality reduction of data was performed by PCA unsupervised algorithm, and visualization was carried out to present the repeatability between groups.

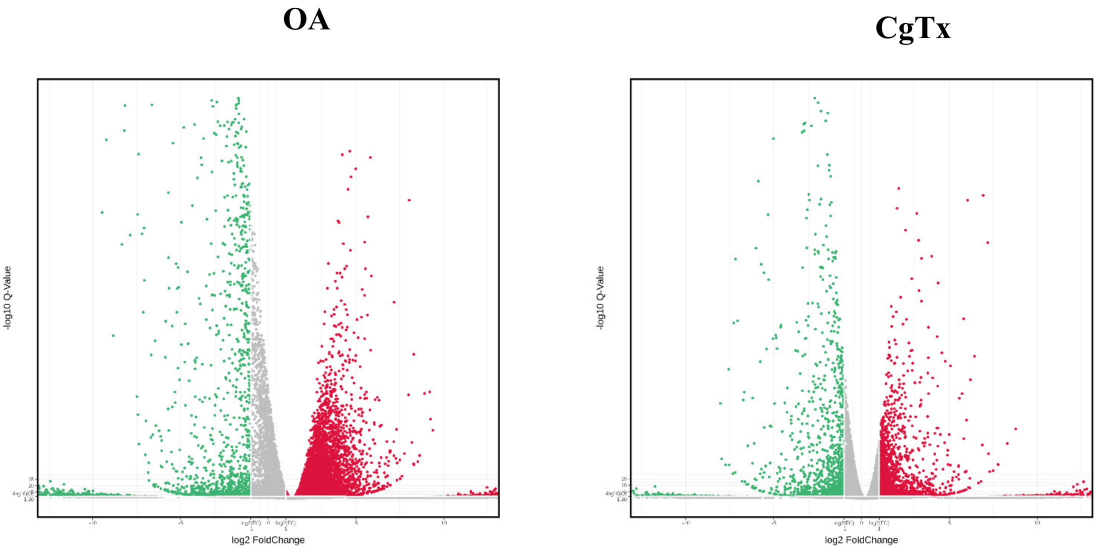

Figure S12. Volcanic map. The difference mRNA was visualized.

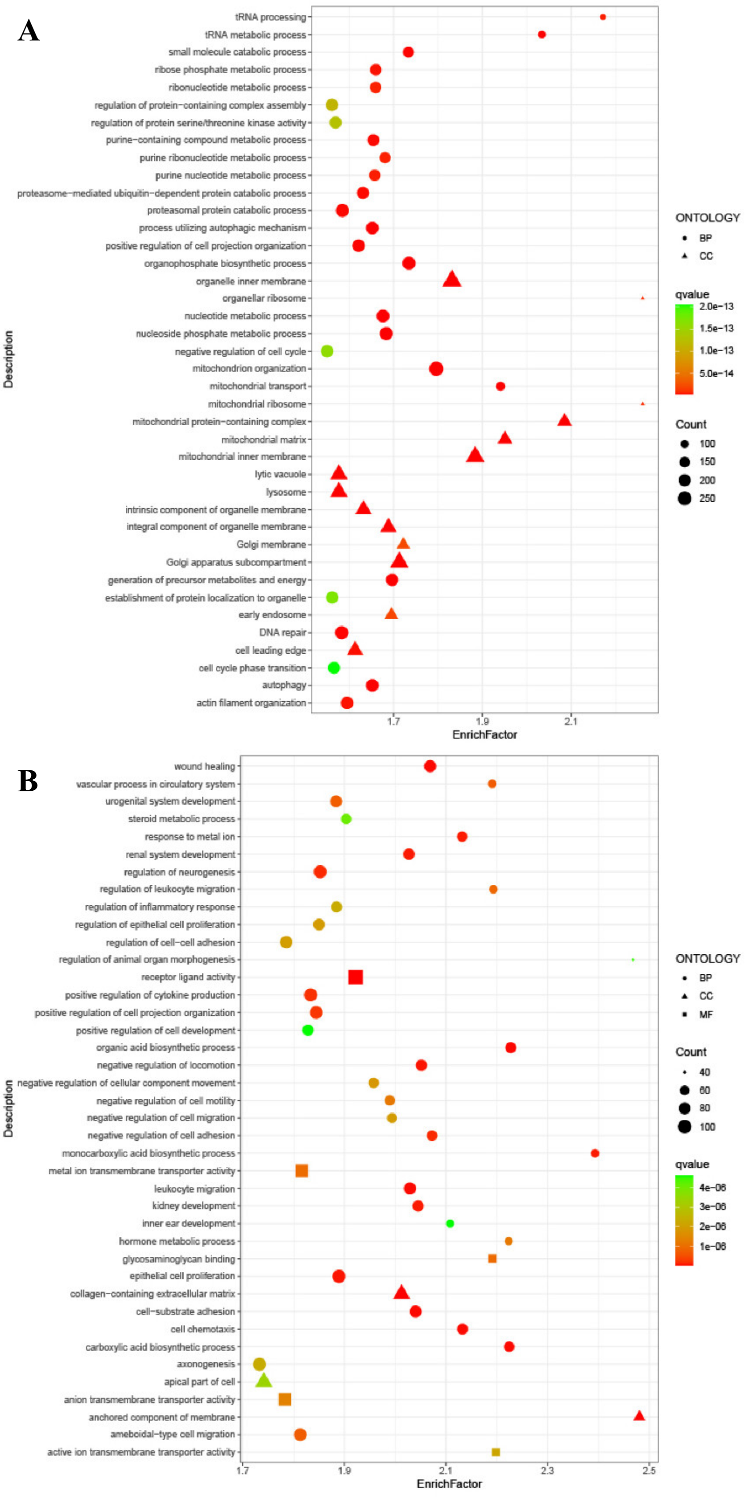
A
tRNA processing
tRNA metabolic process
small molecule catabolic process
ribose phosphate metabolic process
ribonucleotide metabolic process
regulation of protein−containing complex assembly
regulation of protein serine/threonine kinase activity
purine−containing compound metabolic process
purine ribonucleotide metabolic process
purine nucleotide metabolic process
proteasome−mediated ubiquitin−dependent protein catabolic process
proteasomal protein catabolic process
process utilizing autophagic mechanism
positive regulation of cell projection organization
organophosphate biosynthetic process
organelle inner membrane
organellar ribosome
nucleotide metabolic process
nucleoside phosphate metabolic process
negative regulation of cell cycle
mitochondrion organization
mitochondrial transport
mitochondrial ribosome
mitochondrial protein−containing complex
mitochondrial matrix
mitochondrial inner membrane
lytic vacuole
lysosome
intrinsic component of organelle membrane
integral component of organelle membrane
Golgi membrane
Golgi apparatus subcompartment
generation of precursor metabolites and energy
establishment of protein localization to organelle
early endosome
DNA repair
cell leading edge
cell cycle phase transition
autophagy
actin filament organization
Description
1.7
1.9
2.1
EnrichFactor
ONTOLOGY
BP
CC
qvalue
2.0e−13
1.5e−13
1.0e−13
5.0e−14
Count
100
150
200
250
B
wound healing
vascular process in circulatory system
urogenital system development
steroid metabolic process
response to metal ion
renal system development
regulation of neurogenesis
regulation of leukocyte migration
regulation of inflammatory response
regulation of epithelial cell proliferation
regulation of cell−cell adhesion
regulation of animal organ morphogenesis
receptor ligand activity
positive regulation of cytokine production
positive regulation of cell projection organization
positive regulation of cell development
organic acid biosynthetic process
negative regulation of locomotion
negative regulation of cellular component movement
negative regulation of cell motility
negative regulation of cell migration
negative regulation of cell adhesion
monocarboxylic acid biosynthetic process
metal ion transmembrane transporter activity
leukocyte migration
kidney development
inner ear development
hormone metabolic process
glycosaminoglycan binding
epithelial cell proliferation
collagen−containing extracellular matrix
cell−substrate adhesion
cell chemotaxis
carboxylic acid biosynthetic process
axonogenesis
apical part of cell
anion transmembrane transporter activity
anchored component of membrane
ameboidal−type cell migration
active ion transmembrane transporter activity
Description
1.7
1.9
2.1
2.3
2.5
EnrichFactor
ONTOLOGY
BP
CC
MF
Count
40
60
80
100
qvalue
4e−06
3e−06
2e−06
1e−06

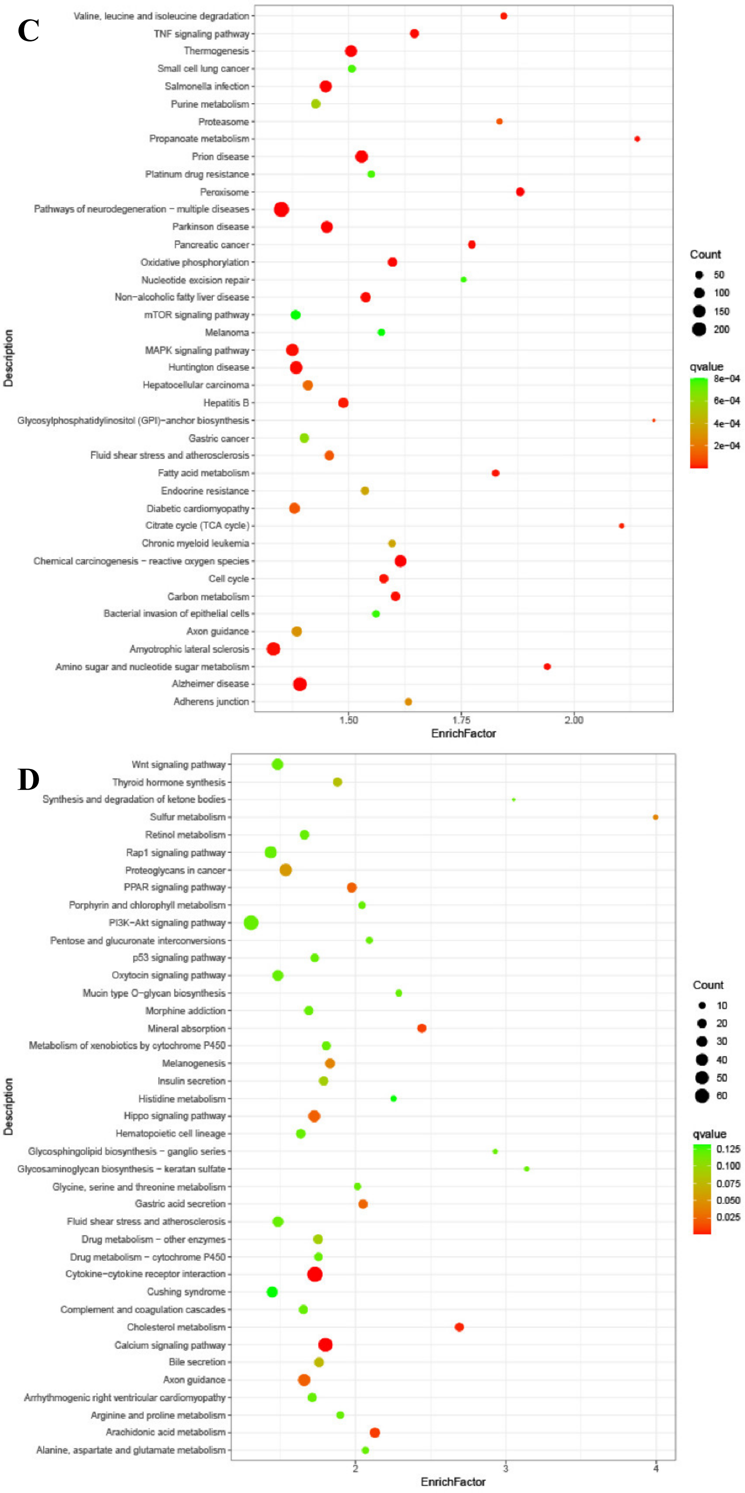

Figure S13. Mouse intestinal organoids were subcultured for 14 days, the differential miRNA target genes were analysed by GO and pathway with the assistance of R packet. GO refers to the international standard classification system of gene function. GO consists of three parts: molecular function, biological process and cellular composition. Through GO enrichment analysis, it is possible to identify not only the important function that leads to the change of traits but also the corresponding genes. KEGG is the bioinformatics resource required to understand biological functions from a genomic perspective. As a multispecies, integrated resource consisting of genomics, chemistry and network

information, it cross-references a number of external databases, including a complete set of construction modules and wiring diagrams to represent cell functions, A and 2 days after adding OA, the concentration was 5 μM, GO analysis; B 2 days after adding CgTx, the concentration was 4 μM, GO analysis; mouse intestinal organoids were subcultured for 14 days, C 2 days after adding OA, the concentration was 5 μM, KEGG analysis; D 2 days after adding CgTx, the concentration was 4 μM, KEGG analysis.

GSEA Report for Dataset B_VS_A_data

Enrichment in phenotype: B (3 samples)

- 753 / 1948 gene sets are upregulated in phenotype **B**
- 0 gene sets are significant at FDR < 25%
- 42 gene sets are significantly enriched at nominal pvalue < 1%
- 42 gene sets are significantly enriched at nominal pvalue < 5%
- Snapshot of enrichment results
- Detailed enrichment results in html format
- Detailed enrichment results in TSV format (tab delimited text)
- Guide to interpret results

Enrichment in phenotype: A (3 samples)

- 1195 / 1948 gene sets are upregulated in phenotype **A**
- 8 gene sets are significantly enriched at FDR < 25%
- 140 gene sets are significantly enriched at nominal pvalue < 1%
- 140 gene sets are significantly enriched at nominal pvalue < 5%
- Snapshot of enrichment results
- Detailed enrichment results in html format
- Detailed enrichment results in TSV format (tab delimited text)
- Guide to interpret results

Dataset details

- The dataset has 7970 features (genes)
- No probe set => gene symbol collapsing was requested, so all 7970 features were used

Gene set details

- Gene set size filters (min=5, max=10000) resulted in filtering out 284 / 2232 gene sets
- The remaining 1948 gene sets were used in the analysis
- List of gene sets used and their sizes (restricted to features in the specified dataset)

Gene markers for the B *versus* A comparison

Figure S14. The differential genes of OA vs_Control were analyzed by pathway, and the related pathways of differential genes were predicted. According to the enrichment analysis, the method adopted was fisher accurate inspection, and the data packet was clusterProfiler, from R/bioconductor.

GSEA Report for Dataset C_VS_A_data

Enrichment in phenotype: C (3 samples)

- 560 / 1151 gene sets are upregulated in phenotype **C**
- 0 gene sets are significant at FDR < 25%
- 65 gene sets are significantly enriched at nominal pvalue < 1%
- 65 gene sets are significantly enriched at nominal pvalue < 5%
- Snapshot of enrichment results
- Detailed enrichment results in html format
- Detailed enrichment results in TSV format (tab delimited text)
- Guide to interpret results

Enrichment in phenotype: A (3 samples)

- 591 / 1151 gene sets are upregulated in phenotype **A**
- 1 gene sets are significantly enriched at FDR < 25%
- 45 gene sets are significantly enriched at nominal pvalue < 1%
- 45 gene sets are significantly enriched at nominal pvalue < 5%
- Snapshot of enrichment results
- Detailed enrichment results in html format
- Detailed enrichment results in TSV format (tab delimited text)
- Guide to interpret results

Dataset details

- The dataset has 2985 features (genes)
- No probe set => gene symbol collapsing was requested, so all 2985 features were used

Gene set details

- Gene set size filters (min=5, max=10000) resulted in filtering out 1081 / 2232 gene sets
- The remaining 1151 gene sets were used in the analysis
- List of gene sets used and their sizes (restricted to features in the specified dataset)

Gene markers for the C *versus* A comparison

Figure S15. The differential genes of CgTx vs_Control were analyzed by pathway, and the related pathways of differential genes were predicted. According to the enrichment analysis, the method adopted was fisher accurate inspection, and the data packet was clusterProfiler, from R/bioconductor.

Table S1. Sequencing data quantity and quality control.

##	External sample number	data output （Gb）	Reads（M）	Q30（%）
## 1	22026-7-B2	6.493	43.29	90.69
## 2	22026-7-B3	8.057	53.71	91.32
## 3	22026-7-B1	7.343	48.95	91.09
## 4	22026-7-A1	8.51	56.73	93.91
## 5	22026-7-A2	9.726	64.84	93.65
## 6	22026-7-A3	8.179	54.53	93.76
## 7	22026-7-C1	7.176	47.85	93.77
## 8	22026-7-C2	7.984	53.23	93.68
## 9	22026-7-C3	8.273	55.16	94.09

Table S2. Quality and error rate comparison table.

##	Error rate of Phred score	sequencing (E)	Q value
## 1	10	10%	Q10
## 2	20	1%	Q20
## 3	30	0.10%	Q30
## 4	40(max Q)	0.01%	Q40

Table S3. Statistical results after data filtering.

##	Sample	RawReads	CleanReads	CleanRatio	rmrRNAreads	rRNA_Ratio
## 1	A1	56,733,672	56,629,286	99.82%	56,520,858	0.19%
## 2	A2	64,842,264	64,714,004	99.8%	64,606,912	0.17%
## 3	A3	54,531,554	54,433,752	99.82%	54,312,786	0.22%
## 4	B1	48,959,732	48,860,008	99.8%	48,673,972	0.38%
## 5	B2	43,290,206	43,196,540	99.78%	43,043,604	0.35%
## 6	B3	53,719,508	53,620,804	99.82%	53,426,910	0.36%
## 7	C1	47,846,408	47,770,716	99.84%	47,696,248	0.16%
## 8	C2	53,227,284	53,144,582	99.84%	53,079,874	0.12%
## 9	C3	55,158,816	55,056,522	99.81%	54,993,428	0.11%

Table S4. Mapping statistical results.

##	Sample Name	ALL Reads	Mapped Reads	Mapped Unique Reads	Mapped Multi Reads
## 1	A1	56,520,858	53,835,793	47,864,931	5,970,862
## 2	A2	64,606,912	60,981,492	54,003,461	6,978,031
## 3	A3	54,312,786	51,815,985	46,247,180	5,568,805
## 4	B1	48,673,972	41,797,571	26,926,998	14,870,573
## 5	B2	43,043,604	36,908,782	22,437,032	14,471,750
## 6	B3	53,426,910	46,203,745	28,642,362	17,561,383
## 7	C1	47,696,248	44,521,385	40,049,694	4,471,691
## 8	C2	53,079,874	49,669,232	44,683,208	4,986,024
## 9	C3	54,993,428	49,343,776	44,516,069	4,827,707

##	Alignment Rate
## 1	95.25%
## 2	94.39%
## 3	95.40%
## 4	85.87%
## 5	85.75%
## 6	86.48%
## 7	93.34%
## 8	93.57%
## 9	89.73%

Table S5. GSEA of mouse intestinal organoids were subcultured for 14 days and added OA for 2 days, concentration 5 μM; top 10 pathway with positive correlation with OA.

NAME	SIZE	ES	NES	NOM p-val	FDR q-val	FWER p-val	RANK AT MAX	LEADING EDGE
BIOCARTA_GCR_PATHWAY	11	0.49453455	1.8651773	0	0.558652	0.416	4033	tags=100%, list=51%, signal=202%
REACTOME_ARMS_MEDIATED_ACTIVATION	7	0.513226	1.7686964	0	1	0.705	2396	tags=86%, list=30%, signal=122%
REACTOME_MRNA_SPLICING_MINOR_PATHWAY	25	0.31642532	1.6650683	0	1	0.906	5455	tags=100%, list=68%, signal=316%
PID_CXCR4_PATHWAY	57	0.20027506	1.6257151	0	1	0.966	5231	tags=89%, list=66%, signal=258%
REACTOME_PROLONGED_ERK_ACTIVATION_EVENTS	11	0.3507725	1.6213895	0	1	0.966	2396	tags=73%, list=30%, signal=104%
REACTOME_CTLA4_INHIBITORY_SIGNALING	8	0.44875655	1.5325488	0	1	1	4396	tags=100%, list=55%, signal=223%
BIOCARTA_CDK5_PATHWAY	11	0.29562044	1.5234358	0	1	1	350	tags=27%, list=4%, signal=28%
REACTOME_REGULATION_OF_IFNA_SIGNALING	10	0.35213578	1.5083532	0	1	1	5166	tags=100%, list=65%, signal=284%

PID_P38_MKK3_6PATHWAY	15	0.25945166	1.4905676	0	1	1	3988	tags=80%, list=50%, signal=160%
REACTOME_REGULATION_OF_LOCALIZATION_OF_FOXO_TRANSCRIPTION_FACTORS	6	0.494224	1.4875777	0	1	1	4033	tags=100%, list=51%, signal=202%

Table S6. GSEA of mouse intestinal organoids were subcultured for 14 days and added OA for 2 days, concentration 5 μM; top 10 pathway with negative correlation with OA.

NAME	SIZE	ES	NES	NOM p-val	FDR q-val	FWER p-val	RANK AT MAX	LEADING EDGE
REACTOME_PRE_NOTCH_PROCESSING_IN_THE_ENDOPLASMIC_RETICULUM	5	-0.62724227	-2.09512	0	0.045	0	1960	tags=80%, list=25%, signal=106%
KEGG_RIBOFLAVIN_METABOLISM	11	-0.52075285	-1.99508	0	0.088805	0.126	3009	tags=82%, list=38%, signal=131%
REACTOME_DISEASES_OF_METABOLISM	149	-0.25797108	-1.98037	0	0.074204	0.126	2285	tags=44%, list=29%, signal=60%
KEGG_DORSO_VENTRAL_AXIS_FORMATION	14	-0.44836396	-1.93558	0	0.075496	0.126	2554	tags=64%, list=32%, signal=94%
REACTOME_METABOLISM_OF_VITAMINS_AND_COFACTORS	120	-0.28042835	-1.93073	0	0.069397	0.126	2309	tags=48%, list=29%, signal=66%
REACTOME_ERBB2_ACTIVATES_PTK6_SIGNALING	5	-0.61160076	-1.85775	0	0.132711	0.342	1595	tags=80%, list=20%, signal=100%
KEGG_GLYCOSPHINGOLIPID_BIOSYNTHESIS_LACTO_AND_NEOLACTO_SERIES	9	-0.53868747	-1.85416	0	0.136956	0.399	3551	tags=89%, list=45%, signal=160%
REACTOME_METABOLISM_OF_WATER_SOLUBLE_VITAMINS_AND_COFACTORS	78	-0.33920977	-1.83961	0	0.158573	0.514	2286	tags=54%, list=29%, signal=75%

REACTOME_ETHANOL_OXIDATION	10	-0.42225832	-1.78382	0	0.274716	0.756	590	tags=40%, list=7%, signal=43%
REACTOME_CREATINE_METABOLISM	5	-0.62988377	-1.76998	0	0.305683	0.756	479	tags=60%, list=6%, signal=64%

Table S7. GSEA of mouse intestinal organoids were subcultured for 14 days and added CgTx for 2 days, concentration 4 μM; top 10 pathway with positive correlation with CgTx.

NAME	SIZE	ES	NES	NOM p-val	FDR q-val	FWER p-val	RANK AT MAX	LEADING EDGE
PID_E2F_PATHWAY	21	0.38214323	1.6896714	0	1	0.52	318	tags=33%, list=11%, signal=37%
REACTOME_DOWNREGULATION_OF_TGF_BETA_RECEPTOR_SIGNALING	7	0.5226088	1.6890312	0	0.8109343	0.618	353	tags=57%, list=12%, signal=65%
REACTOME_RETINOID_CYCLE_DISEASE_EVENTS	6	0.61967325	1.644516	0	0.9908863	0.948	191	tags=50%, list=6%, signal=53%
REACTOME_EGFR_DOWNREGULATION	5	0.56453055	1.6302446	0	0.8847057	0.948	23	tags=40%, list=1%, signal=40%
REACTOME_TGF_BETA_RECEPTOR_SIGNALING_ACTIVATES_SMADS	11	0.37205595	1.626635	0	0.7434853	0.948	353	tags=36%, list=12%, signal=41%
KEGG_BUTANOATE_METABOLISM	13	0.3685832	1.6000984	0	0.8087791	1	449	tags=46%, list=15%, signal=54%
REACTOME_ABC_FAMILY_PROTEINS_MEDIATED_TRANSPORT	20	0.2989124	1.5532861	0	1	1	558	tags=40%, list=19%, signal=49%
REACTOME_PEPTIDE_HORMONE_METABOLISM	19	0.42398852	1.5530871	0	1	1	318	tags=37%, list=11%, signal=41%

PID_TRKR_PATHWAY	20	0.34202713	1.5374722	0	1	1	245	tags=30%, list=8%, signal=32%
REACTOME_POST_TRANSLATIONAL_PROTEIN_MODIFICATION	276	0.1847598	1.4960033	0	1	1	572	tags=28%, list=19%, signal=31%

Table S8. GSEA of mouse intestinal organoids were subcultured for 14 days and added CgTx for 2 days, concentration 4 μM; top 10 pathway with negative correlation with CgTx.

NAME	SIZE	ES	NES	NOM p-val	FDR q-val	FWER p-val	RANK AT MAX	LEADING EDGE
REACTOME_LONG_TERM_POTENTIATION	7	-0.6880439	-1.783636	0	0.504974	0.235	175	tags=57%, list=6%, signal=61%
REACTOME_CREB1_PHOSPHORYLATION_THROUGH_ NMDA_RECEPTOR_MEDIATED_ACTIVATION_OF_RAS_SIGNALING	6	-0.67519295	-1.781969	0	0.2973932	0.285	94	tags=50%, list=3%, signal=52%
REACTOME_UNBLOCKING_OF_NMDA_RECEPTORS_GLUTAMATE _BINDING_AND_ACTIVATION	6	-0.66089743	-1.779965	0	0.2149289	0.285	107	tags=50%, list=4%, signal=52%
KEGG_PYRIMIDINE_METABOLISM	12	-0.5398126	-1.698	0	0.3687008	0.715	564	tags=58%, list=19%, signal=72%
BIOCARTA_EXTRINSIC_PATHWAY	5	-0.5875213	-1.674801	0	0.4591327	0.827	259	tags=60%, list=9%, signal=66%

KEGG_REGULATION_OF_ACTIN_CYTOSKELETON	45	-0.3084364	-1.597941	0	0.8610355	0.937	350	tags=29%, list=12%, signal=32%
KEGG_NICOTINATE_AND_NICOTINAMIDE_METABOLISM	11	-0.3902914	-1.585324	0	0.8484871	1	369	tags=36%, list=12%, signal=41%
REACTOME_COMMON_PATHWAY_OF_FIBRIN_CLOT_FORMATION	12	-0.4406859	-1.56492	0.2007874	0.9533182	1	338	tags=42%, list=11%, signal=47%
KEGG_GLYCEROLIPID_METABOLISM	15	-0.35926643	-1.550601	0.1165644	1	1	330	tags=33%, list=11%, signal=37%
PID_FCER1_PATHWAY	9	-0.45894766	-1.541932	0	1	1	779	tags=56%, list=26%, signal=75%
